# Review of *Planning for uncertainty: living wills and other advance directives for you and your family*, 2nd edition by David John Doukas, M.D., and William Reichel, M.D

**DOI:** 10.1186/1747-5341-3-13

**Published:** 2008-05-22

**Authors:** Ellen W Bernal

**Affiliations:** 1 St. Vincent Mercy Medical Center, Toledo, OH 43608, USA

## Abstract

Advance directives are useful ways to express one's wishes about end of life care, but even now most people have not completed one of the documents. David Doukas and William Reichel strongly encourage planning for end of life care. Although *Planning for Uncertainty *is at times fairly abstract for the general reader, it does provide useful background and practical steps.

## 

During the 1970's and 1980's legal, ethical and medical communities in the United States began to articulate the right of the adult, informed patient with decisional capacity to accept or decline life-sustaining treatment [1,2]. A series of widely-publicized clinical cases led to critical examination of the assumption that patients must accept any available form of treatment [3,4]. The case of Nancy Cruzan in Missouri was especially influential. In the winter of 1983, Ms. Cruzan sustained an anoxic brain injury as the result of an automobile accident. She was resuscitated at the scene, taken to a hospital and placed on ventilator support. She was eventually able to breathe without the support of a respirator, but neurologically she lapsed into a persistent vegetative state. After she had spent seven years in an extended care facility, maintained by nursing care and a feeding tube, Nancy's parents petitioned the court for the right to withdraw medically supplied nutrition and hydration. Their petition was first granted in probate court, but the Missouri Supreme Court, on appeal, held that there must be clear and convincing evidence of the patient's wishes before life-sustaining treatment can be withdrawn. On appeal, the United States Supreme Court decided in 1990 that incompetent patients have the right to refuse life-sustaining treatment, including medically supplied nutrition and hydration, provided there is clear and convincing evidence of their wishes. Each state was at liberty to set its own standards for clear and convincing evidence. Several months later, friends who had learned of Nancy Cruzan's situation provided additional information about her previously stated wishes and the feeding tube was withdrawn.

The United States Congress passed the Patient Self-Determination Act (P.S.D.A.) in November, 1990 [5]. Hospitals in the United States were now required by federal law and accreditation organizations to ask every patient on admission whether they have completed an advance directive (a living will or a durable power of attorney for health care document) and if so, to request that they bring a copy to the hospital. If the patient does not have an advance directive but would like more information, hospitals must also provide the information.

The majority of states also developed advance directive laws. There are many websites that provide details and some feature on-line completion and registration of the documents^a ^[6]. Across the United States, communities have developed programs that support completion of advance directive documents, as well as promote "advance care planning," a wider exploration of life values and conversations with loved ones as essential steps in decision making [7]. Despite all this activity, estimates suggest that only 25% to 50% of adults in the United States have completed an advance directive [8-10].

The recent St. Petersburg, Florida case of Terri Schindler Schiavo was further indication of public ambivalence about advance directives in the United States. In 1990, at 27 years of age, Terri Schiavo collapsed in her home and experienced cardiac and respiratory arrest. Like Nancy Cruzan, Terri Schiavo suffered extensive brain damage, lapsed into a persistent vegetative state, and was maintained on a feeding tube in an extended care facility [11]. In 1998, Terri's husband and guardian, Michael Schiavo, petitioned the Pinellas County Circuit Court for the right to remove her feeding tube. However, Terri's parents, Robert and Mary Schindler, were adamantly opposed. A complex series of legal appeals, acts of congress, federal legislation and international controversy continued until March, 2005 when Schiavo's feeding tube was withdrawn. Clearly, issues believed to be resolved after the case of Nancy Cruzan are still unsettled.

Family physicians Dr. John Doukas and Dr. William Reichel published the first edition of *Planning for Uncertainty: a Guide to Living Wills and Other Advance Directives for Health Care in 1993*. Their intention was to educate patients and families about the importance of end of life planning and the need to develop advance directives. The 2007 edition updates and restates information in light of new developments such as the Terri Schiavo case. The authors' continued focus on advance care planning is also based on difficult patient and family situations they have encountered over the years; and indeed the book is framed as a series of questions and answers.

As Dr. Doukas and Dr. Reichel imply, it is a challenge to motivate people to complete advance directives – a topic most would prefer to avoid. Further, even though the right of patients to make their own health care decisions has been well established in the United States, federal and state laws covering advance directives are confusing and complex. How, exactly, are we to express our wishes in a "clear and convincing" manner?

*Planning for Uncertainty *seeks to bridge this gap. The book has eight chapters, a useful appendix with state-by-state internet links to advance directive information, a sample Values History and Advance Directive forms. An index provides ready access to specific topics.

The book's introduction uses the Schiavo case as a reason why everyone should give thought to advance care planning. It includes brief definitions of living wills, health care powers of attorney, and the importance of sharing perspectives with one's doctor and family.

Chapter 1 goes over what patients admitted to a hospital in the United States may expect as a result of the Patient Self-Determination Act. The authors express the hope that the law's implementation will encourage careful conversations about advance directives, but are concerned that all too often, discussions between patients and doctors will not occur.

The book proceeds to give readers tools needed to facilitate these conversations. Chapter 2 differentiates between beneficial and nonbeneficial treatments and explains the doctor's professional prerogative not to provide medically nonbeneficial measures. Chapter 3 follows with the ethical principles of autonomy, beneficence, and justice. The chapter includes one of the most useful features of the book: specific questions that a patient should consider asking the doctor if the information has not already been given; for example, the risks and benefits of a proposed therapy and whether there are alternate treatments. These questions would be helpful to any patient; not just those in the United States.

After a brief section on values in Chapter 4, Chapter 5 provides more details on advance directives. Chapter 6 returns to values again, this time the Values History as a supplement to advance directives. From an organizational standpoint, it might have been better to combine the values discussions into one chapter. Chapter 7 discusses factors in choosing a proxy, and Chapter 8 recommends a process for completing an advance directive. It would have been helpful to include a bulleted list of process steps.

Overall, *Planning for Uncertainty *contains much helpful information about advance directives and advance care planning, with a few minor exceptions. The distinctions between "active euthanasia" and "passive euthanasia" are confusing; phrases such as "assisted suicide," "changing goals of care from primarily curative to comfort," and "allowing nature to take its course" are more useful. Also, as noted previously, in some instances the book's organization might be improved. Chapter 6, "The Values History: Defining Your Health Care Values" provides clear definitions of ventilators, CPR, and medically supplied nutrition and hydration, but "heroic measures" and a previous discussion of nutrition and hydration are found in the Introduction, and "assisted suicide" is in Chapter 8.

A more important concern is the accessibility of the book's information to patients or families with a high school education or below. The ideas and language used are complex and abstract. To be sure, bioethics terminology is often abstract, but information intended for a wider audience needs to be straightforward and practical. *Planning for Uncertainty *would not be among the first choices for the general public. Short booklets that help people "walk through" their values, beliefs and health care choices are more practical. The Ohio End of Life Collaborative booklet, "Conversations that Light the Way," offers a series of questions and hypothetical situations that would be useful for many persons within the United States and internationally [12]. It would be a simple matter to provide information on advance directive documents appropriate to the specific setting [13]. Still, for those needing more detail and reflection, *Planning for Uncertainty *is a good resource.

## About the author

EWB, PhD is Director of Ethics, St. Vincent Mercy Medical Center, Toledo, Ohio. She provides ethics consultation, teaches medical professionals, and chairs two Institutional Review Boards. Her publications include End of life decisions: family views on advance directives, *Am J Hosp Palliat Care *2007 [3], and Listening to the husband in *Complex Ethics Consultations: Cases that Haunt Us *[14]. She is past president of the Bioethics Network of Ohio.

## Competing interests

The author declares that they have no competing interests.
